# Quality of maize for sale in markets in Benin and Niger

**DOI:** 10.1016/j.jspr.2017.02.001

**Published:** 2017-03

**Authors:** O.N. Bakoye, I.B. Baoua, H. Seyni, L. Amadou, L.L. Murdock, D. Baributsa

**Affiliations:** a Institut National de la Recherche Agronomique du Niger (INRAN), BP 240 Maradi, Niger; b Université de Maradi, BP 465 Maradi, Niger; c Université Abdou Moumouni de Niamey, BP 10662 Niamey, Niger; d Department of Entomology, Purdue University, West Lafayette, IN 47907, USA

**Keywords:** Maize, Infestation, Aflatoxin, Market, Trader, Insect

## Abstract

A follow-up study on the quality of maize for sale in West African public markets was carried out in Benin and Niger from August 15–28, 2013. Complementing the earlier study, this present assessment included not only retailers but also wholesalers and maize producers. Samples were evaluated for parameters related to the physical quality of the maize and for aflatoxin contamination. Most maize value chain actors process their offered grain using traditional methods for threshing, winnowing and drying. Maize for sale in the markets surveyed had an average moisture content ranging between 12 and 14%. Non-grain impurities amounted to 0–2.3% while mouldy grains ranged between 0.2 and 0.8%. The impurity level in grain was three times higher among wholesalers compared to retailers and producers. An insect pest, the Larger Grain Borer (*Prostephanus truncatus* (Horn) was found only in Benin but *Sitophilus zeamais* Motschulsky, *Cryptolestes ferrugineus* Stephens, and *Tribolium castaneum* Herbst, were present in maize for sale in the markets in of both countries. Insect pest frequency was 16 times higher in wholesalers' grain compared to that of retailers and producers. Aflatoxin levels exceeding the accepted standard of 20 ppb were noted in markets in both countries. The highest proportion of aflatoxin-contaminated maize was in wholesalers’ grain in Malanville market.

## Highlights

• We assessed the quality of maize for sale in local markets in Benin and Niger.
• Wholesalers' grain has the highest levels of non-grain impurities.
• Grain from retailers and producers had fewer insects than that from wholesalers.
• Most maize samples were safely dry, with moisture levels of 12–14 percent.
• Some maize samples has aflatoxin above the 20 ppb level.

## 1 Introduction

In Africa, maize is a primary food crop for human consumption. In the Sahelian zone, it is consumed in the form of pasta, porridge, pancakes, couscous or grilled (Rouanet, 1984). In Benin, maize production was estimated to be 1.1 million tons in 2012/2013 (Gain Report, 2013), Niger produces comparatively little maize, with annual production estimated at around 7610 tons in 2012/2013 (M.A, 2012). There is need to intensify maize production in Niger, especially in the regions of Agadez, Diffa and Tillabery, which have the lowest yields. Given its maize supply deficit, Niger imports about 45,000 tonnes from Benin, Nigeria, Togo, Ghana, and Burkina Faso (INS-Niger, 2011). Despite its importance, maize faces many problems from production to storage. These are mainly related to the traditional harvesting, drying and storage processes that favour the presence of impurities in the grain and that also expose it to insect pests. In parts of Africa *Prostephanus truncatus* (Horn), the Large Grain Borer (LGB), is a major pest of maize. Losses can reach between 15 and 35% after six to eight months of storage (Chittenden, 1895, Back and Cotton, 1922, Haubruge and Gaspar, 1990, Golob and Hodges, 1982).

Other important insect pests of stored maize include *Sitophilus zeamais* Motschulsky, *Rhizopertha dominica* F., *Tribolium castaneum* Herbst, *Tribolium confusum* Jacquelin du Val (Ortega, 1987, Jacobs and Calvin, 2001, Throne, 1994, Markham et al., 1994).

Aflatoxin contamination is also a major constraint to the utilization and value of maize. *Aspergillus flavus* is one of the most common fungi that produces aflatoxin B1, recognized as a potential carcinogen by the International Agency for Research on Cancer (IARC (International Agency for Research on Cancer) , 1993). The main factors that influence the development of fungi in cereals are moisture content and storage temperature (Mitchell et al., 2004). In the tropics, the temperature is almost always favorable to the development of fungi, making moisture content the main controllable variable affecting the invasion and the growth of fungi (Northolt et al., 1976). Aflatoxin B1 is a threat to human health and food security but also an economic concern to maize traders. Most developed countries have regulations fixing the allowable levels in food for humans and livestock. In the USA the accepted level for human consumption is 20 ppb (Wood, 1992) and for the European Union, the maximum acceptable total aflatoxin (B1, B2, G1 and G2) in nuts, dried fruits, cereals and spices is 4–15 ppb (http://www.foodsafetywatch.org/factsheets/aflatoxins/).

These standards hurt the economies of developing countries, especially those that depend on the export of agricultural products to Europe. According to Miller (1996), 40% of maize produced in developing countries contains some level of aflatoxin. In a recent study only 4.6% of maize samples from four West African countries had levels above 20 ppb (Baoua et al., 2016). In Benin, maize produced for human consumption is estimated to be 30% of total crop production (Allogni et al., 2010).

There is little up-to-date information in relation to the quality of maize sold in West African rural markets. This study provides information on post-harvest practices and phytosanitary quality of corn in some rural markets of Benin and Niger and complements our earlier study (Baoua et al., 2016). In this study we worked with traders, growers and market retailers of maize, while the previous study focused on retailers.

## 2 Materials and methods

**Fig. 1:**
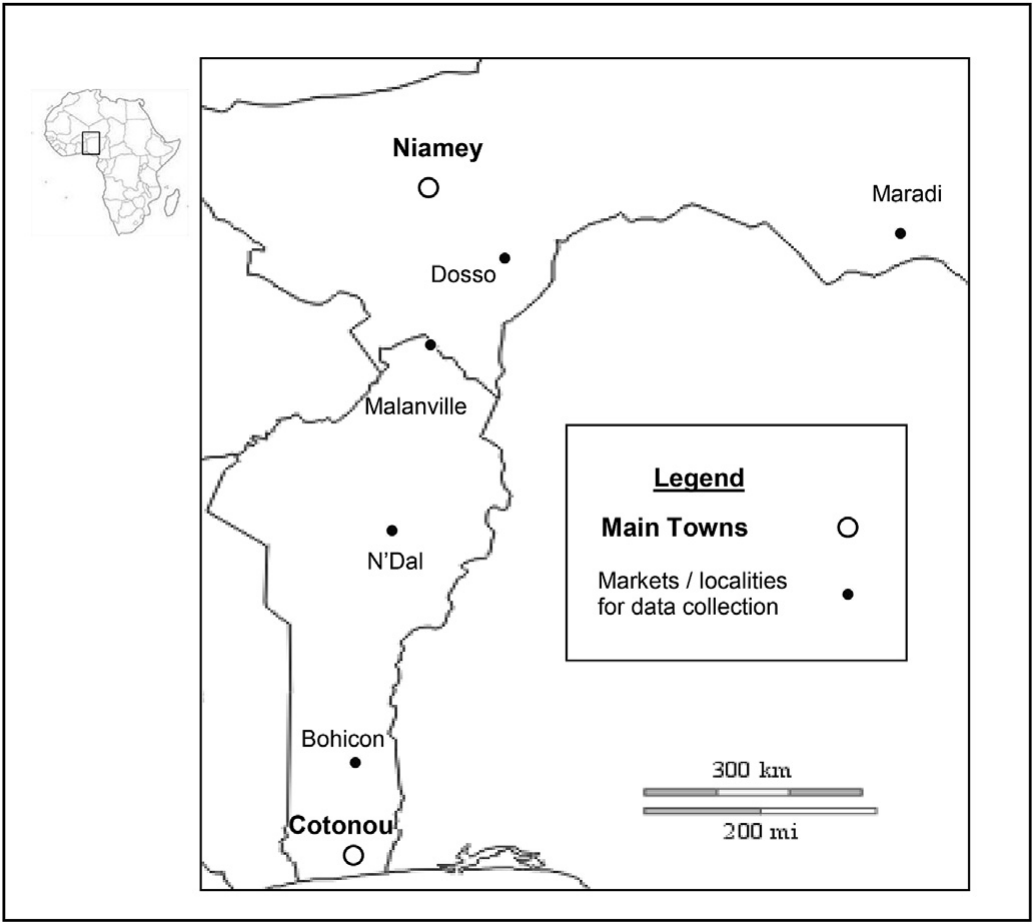
Maize study localities in Benin and Niger.

We conducted our study in five grain markets in Benin and Niger from August 15–28, 2013 (Fig. 1). The annual temperature ranged from 25 °C to 40 °C and the relative humidity between 30% and 90% in the study countries. We focused on wholesalers and retailers in urban markets and producers located near the major urban centers. The Benin markets included: (1) Bohicon, the largest city in the southern forest area of Benin, located 200 km north of Cotonou; (2) N'Dali, located 50 km north of Parakou in the maize-producing zone, and (3) Malanville, which has an international grain market located at the border with Niger and Nigeria. In Niger the study focused on wholesalers and retailers and was carried out in the urban markets of Dosso, located 170 km north of Malanville, and of Maradi, 550 km east of Dosso. Nigerien producers were not involved in the study because they mostly sell only fresh maize to be grilled or boiled in urban centers.

Respondents were selected randomly. Interviews were carried out at the interviewees’ places of work and began with an explanation to them of the reasons for the study. The questionnaire focused on maize post-harvest management processes (drying, threshing, cleaning and winnowing) carried out before the crop storage. Grain moisture content was assessed using a Dickey-John (Dickey-John mini GAC plus Auburn, IL, USA) (http://www.dickey-john.com/product/mini-gac/) grain moisture meter after collecting and measuring three samples from batches of maize being stored by the interviewees. A sample of 500 g of their stored maize was collected for later assessment of grain quality.

Samples of maize to be assayed for aflatoxin were collected using a fresh pair of disposable Latex gloves for each sample. Approximately 200 g of grain were taken and then separated into two samples of 100 g each. These subsamples were repackaged individually in plastic bags and labeled as to date, location and vendor. One of the bags was submitted for aflatoxin analysis and the second was kept in the freezer for future verification, if necessary. Aflatoxin levels were determined by (ICRISAT) International Crops Research Institute for the Semi-Arid Tropics laboratory in Mali according to the protocols of CIMMYT and ICRISAT using the ELISA procedure (see http://www.icrisat.org/aflatoxin/elisa1.htm for details).

Pest density and grain quality of the maize samples was carried out as follows:

1. A jar was used to collect maize samples from the top, middle and bottom of the storage containers being used by the interviewee. The collected samples were thoroughly mixed and 500 g was saved and labeled for analysis;
2. Mesh sieves of 1 mm, 2 mm and 4.5 mm were used to separate broken grains and residues;
3. Live insects in each sample were identified and counted;
4. Impurities (non-consumables fraction) and mouldy or blackened seeds were separated and weighed to determine its relative proportion compared to the original sample weight.
5. The following formulae were used;
  i. Impurity level % = (weight of impurities/total sample weight) x 100;
  ii. Mouldy grain content % = (weight of mouldy seeds/total sample weight) x 100.

Analysis of variance followed by LSD tests were used to compare means related to moisture levels, percentage of impurities, proportion of mouldy grains, insect density (number of live insects per sample) and aflatoxin level, comparing the different classes of sellers (wholesalers, retailers and producers). Correlation coefficients (Pearson R) between the levels of aflatoxin and the proportion of the impurities, the percentage of mouldy grains and insect density were calculated. Statistical analysis was done with the Statistical Package for the Social Sciences (SPSS) Version 16.0, IBM (Chicago, Illinois).

## 3 Results

We collected 112 maize samples, 90 in Benin and 22 in Niger (Table 1). In Benin, 33 samples were obtained from wholesale traders, 33 others with retailers and 24 from producers. In Niger we collected 11 samples from wholesalers and 11 from retailers. Samples from Malanville, N'Dali, Dosso and Maradi were for the 2012 harvest year and from Bohicon for the 2013 harvest year.

Postharvest practices in use: 1) Drying: all respondents dry their crop in the household yard by exposing it to the sun on bare soil, on the roofs of the houses, on the edges of the paved roads, or in another cleared area. These methods are used for shelled maize. Reported drying times varied from 7 to 15 days; 2) Threshing: all respondents used the traditional method, beating the cobs with clubs or using mortars and pestles; 3) Cleaning and winnowing: Threshed grain is transferred to a basket that is held high and shaken in the breeze to separate grain from impurities.

Harvesting and threshing were done only at the producer level; drying was done by producers, by wholesalers as well as by retailers; cleaning (winnowing and sifting), was often done by retailers.

Moisture content of grain ranged from 11.8% to 22.7% depending on the location and the individual providing the sample of grain. The mean grain moisture content in the three Benin urban markets ranged from 11.8% to 14.1% among wholesalers; from 13.6% to 22.7% among producers and 13.0%–17.5% among retailers. In Niger it ranged from 12.7% to 13.0% for wholesalers in Maradi and 12.8%–13.3% for retailers in Dosso. Mean grain moisture content was similar at Malanville, Dosso and Maradi (Table 2). At N'Dali and Bohicon it was highest with farmers and retailers compared to wholesalers.

Impurity content varied from 0.0 to 6.3% depending on location and participant. The mean was higher among wholesalers compared to retailers and producers in Malanville, N'Dali, and Bohicon. In Dosso, maize samples collected from wholesalers had high impurity levels compared with maize stored by retailers, while in Maradi, impurity levels were similar between wholesalers and retailers. Considering all five localities, the average impurity level observed with wholesalers was more than twice that observed with retailers.

We observed different insect pests in maize in Benin and Niger (Table 3). *P. truncatus* was found only at Malanville and only in grain from wholesalers. Maize weevil (*S. zeamais*) was observed in N'Dali, Dosso and Maradi samples, with higher infestations in grain from wholesalers. For the five sites the mean infestation level among wholesalers was 1.8 ± 0.3 live insects per 500 g of grain, some 18 times higher than observed in retailers' grain. The secondary pests *C. ferrugineus and T. castaneum* were observed at low levels in grain from wholesalers in Malanville, N'Dali, Dosso and Maradi. No primary or secondary insect pests were identified in maize samples collected in Bohicon. The total number of insects per 500 g samples varied from 0 to 10 insects in the different localities and among the different holders of maize. Across localities, the mean number of insects per 500 g was in wholesalers' grain (3.2 ± 0.4), 16 times higher than observed in retailers' grain.

The proportion of mouldy grain ranged from 0.0 to 2.2%. For all five locations it was higher for wholesalers' grain than for retailers' and producers’. It averaged 0.8± 0.1% with wholesalers, at least twice as high as with retailers and four times higher than observed with producers. Out of the 112 samples analyzed, 13 (11.6%) had no detectable aflatoxin contamination; 81 (72.2%) contained between 0.1 and 16.7 ppb, while 18 (16.1%) had from 60.5 to 2579.8 ppb.

The percentage of samples with aflatoxin levels above 20 ppb was 72.2% in Malanville; 5.6% in Bohicon; 25.0% in Dosso and 10.0% in Maradi (Table 4). At N'Dali the 54 samples taken had levels ranging from 0.0 to 16.7 ppb, i.e., all samples were below the 20 ppb USA standard.

Overall, the proportion of samples with greater than 20 ppb aflatoxin was 29.5% with wholesalers; 9.1% with retailers and 4.2% with producers. Among all 68 samples not infested by insects; only one had an aflatoxin level above 20 ppb. However, in the 44 samples infested by insects, 13 exhibited aflatoxin levels above 20 ppb.

The Pearson correlation between aflatoxin level and the other parameters were 11.6% (P = 0.11) with the moisture content of the seeds; 29.4% (P < 0.01) with the percentage of non-consumable fractions; 19.2% (P < 0.05) with the proportion of mouldy seeds and 21.4% (P < 0.05) with the total number of insects observed in 500 g of maize.

## 4 Discussion

Our results shed light on maize quality along the supply chain in Benin and Niger. The postharvest practices and processes used by producers, retailers and wholesalers are substantially traditional. Harvested maize is dried on the ground in the sun; grain is shelled using clubs or mortars; and wind is used for winnowing. None of the 112 participants surveyed used modern mechanical or electrical equipment for shelling and cleaning. According to Armelle and Dominique (2013), whose study was conducted in Benin, these antiquated post-harvest practices are responsible for maize losses on the order of 15–30%. In West Africa, low-cost shellers and mechanical dryers have been developed that are labor-saving and more efficient than traditional methods (Bassey and Schmidt, 1989, Holtzman et al., 1991, Meuser and Smolnik, 1980). Adoption of these technologies would improve the uality of maize by minimizing the exposure of corn on the ground, thereby reducing debris and aflatoxin content.

In Benin, grain moisture levels in maize kept by wholesalers varied from 11.8 to 14.1%, indicating that traders dry their grain well before bagging it for sale. These moisture levels meet the standards of 14% moisture recommended by the World Food Program (WFP) (Hodges and Stathers, 2012). A study conducted in the same area of Benin in June 2012 reported similar maize grain moisture contents of 11–13% (Baoua et al., 2014). At Bohicon in southern Benin moisture contents of grain kept by producers and traders were higher, ranging from 17.5 to 22.7%. This maize was just being dried as it had been recently harvested during the 2013 crop season. In Niger, moisture content ranged from 12.7 to 13.3%. Dosso and Maradi markets are often supplied with maize from Benin and the grain moisture contents observed there are similar to those recorded in Malanville.

Overall, the mean grain moisture content varied between 12.7 and 13.7% for wholesalers and retailers. The moisture levels we observed paralleled those reported by Scott et al. (2014) who have shown that hermetic storage of maize conditioned at 12 or 15% moisture does not allow the development of aflatoxin in laboratory at 26 °C after one and two months storage. The data we collected here on grain moisture in both countries suggests that maize could be safely stored in hermetic containers such as PICS (Purdue Improve Crop Storage) bags.

The impurity levels in wholesalers' grain averaged 2.3%, which is within the standards of 0.2–3.5% specified by AFNOR (2013) (Association Française de Normalisation). In general, wholesalers purchase small quantities of maize from producers to build up their stocks. However, their usual unit of sale is a bag containing 100–120 kg of grain, with the result that in some cases a bag of grain is a mixture of grain from several farmers. Buyers often have no options other than to purchase what it available – which in some cases may be low quality grain. The impurity content of grain sold by retailers was three times lower than that found in wholesalers' grain. Retailers’ maize was usually sold in small quantities to consumers who routinely assure themselves of good grain quality before purchasing. Recognizing this, retailers improve the market value of their product by sorting and cleaning their grain before putting it out for sale.

The proportion of mouldy grains was very low in general, probably due to the low moisture content of the grain, which ranged between 11 and 14% in the five localities. Scott (1957) demonstrated that there is a positive relationship between the level of moisture in foods and microbial growth capacity. Pitt and Miscamble (1995) reported that a minimum of 82% ambient relative humidity at 25 °C is required to stimulate the development of mildew, while it is 87% for the production of aflatoxin. Our results showed that mouldy grain is much less frequent in retailers' grain compared to wholesalers’. This is undoubtedly linked to the regular sorting and cleaning of grain performed by retailers.

Several insect pests of stored products were identified:

1) Larger grain borer (*P. truncatus*): This is a very serious pest of stored maize in many African countries (Chittenden, 1895, Back and Cotton, 1922, Haubruge and Gaspar, 1990, Holst et al., 2000, Vowotor et al., 2005). We found it in Benin but not in Niger. It has been reported in the western part of Niger (Torodi, Say, Niamey and Dosso) but only in response to pheromone trapping (Adda et al., 1996). Infestations of maize grain in markets have not been reported in Niger;
2) Maize weevil (*S. zeamais*) was found in both Niger and Benin. This species is cosmopolitan and tolerant to low humidity and high temperatures (Pacheco and De Paula. 1995). It is also one of the most important pests of maize in Benin as shown by a survey conducted in 52 localities (Togola et al., 2014);
3) We observed C. ferrugineus and T. castaneum in both countries; they are secondary polyphagous pests that cause little damage to maize in stores (Tuff and Telford, 1964, Suresh et al., 2001, Baoua et al., 2016).

The highest densities of insect pests were observed in the stores of wholesalers. Infestations were 16 times smaller in retailers' stocks when compared to wholesalers', while no infestations were discovered in producers’ grain. The likely explanation for these observations is that wholesalers buy grain from several farmers (some of which may already have low infestations) and hold it for several months. Storage allows the insects to reproduce over multiple generations. Our results show strong correlation between the level of insect density and impurities (r = 82%) and between insect density and mouldy grains r = 62.3%. Higher density of insects in stores leads to higher losses. Wholesalers need to become aware that there are numerous technologies available that provide effective and economical control of insects. Among these technologies are low-cost PICS bags as well as other hermetic technologies. Studies conducted in Sub-Saharan Africa have shown that hermetic storage can reduce losses due to insects such as *P. truncatus* and *S. zeamais* by 21.5 percent after 6.5 months storage (Baoua et al., 2014, Njoroge et al., 2014).

Our results provide new information about the presence, levels and distribution of aflatoxin in maize stores in the region. In Niger as well as in Benin some samples had aflatoxin levels above the 20 ppb standard accepted in the USA (Wood, 1992). Maize in the Malanville market exhibited the highest percentage of aflatoxin-contaminated grain (72.2%). Malanville is one of the largest grain markets of Benin and receives large shipments from the surrounding production areas and cooperatives. Mixing stocks from different localities and harvest seasons in addition to the rudimentary postharvest management in use presumably exacerbates aflatoxin contamination. Wholesalers are the most affected by aflatoxin contamination because they buy their grain in small quantities from many different farmers, combine it, store for long periods, and have poor postharvest management practices. In both countries the proportion of samples with unacceptable levels of aflatoxin is 17.5% and 21.1%, respectively, in Benin and Niger. These rates are well below the 55% noted in Kenya from a study conducted with 350 processed corn samples collected at seven markets (Lewis et al., 2005).

Correlations were calculated in attempts to relate sample moisture levels and contamination levels of aflatoxin. In this study the aflatoxin levels were not correlated to grain moisture as all the examined sample contained 12–14% moisture these are below the 15% moisture content subjected to aflatoxin accumulation reported by Williams et al. (2014). The level of mouldy grain, the level of impurities in the grain, and density of insect pests in it were however significantly correlated with levels of aflatoxin, with correlation coefficients ranging between 19.2 and 21.4%. Each of these parameters may contribute a small percentage of contamination by this toxin. The strongest correlation we observed was related to the density of insect pests. Only one of the 68 non-insect-infested maize samples had abnormal levels of aflatoxin while 13 of the 44 infested samples had aflatoxin levels above 20 ppb and considered unacceptable for human consumption. The presence of insects is evidently associated with increased risk of contamination by the toxin. This is in agreement with other studies that have shown that aflatoxin contamination is positively correlated with level of pest infestation (Lillehoj et al., 1975, McMillian, 1987, Lynch and Wilson, 1991, Beti et al., 1995, Hell et al., 2000).

## 5 Conclusion

This study has led to increased knowledge of grain quality along the maize value chain in Benin and Niger, specifically: 1) Maize for sale in markets generally has low moisture levels ranging between 12 and 14% and with fewer impurities present; 2) The highest proportion of contaminated maize grain samples was noted at the Malanville market especially among wholesalers and traders. Considering these results, it is important to encourage the dissemination of good post-harvest management practices that help reduce pest infestation and aflatoxin contamination, in particular the use of hermetic technology for grain storage.

## Figures and Tables

**Table 1:** Number of collected samples per locality and business of owner.

Localities	Owner	Number of collected samples	Total
Malanville	Wholesalers	9	18
	Farmers	0	
	Retailers	9	
N'Dali	Wholesalers	18	54
	Farmers	18	
	Retailers	18	
Bohicon	Wholesalers	6	18
	Farmers	6	
	Retailers	6	
Dosso	Wholesalers	6	12
	Farmers	0	
	Retailers	6	
Maradi	Wholesalers	5	10
	Farmers	0	
	Retailers	5	
Total			**112**

**Table 2:** Moisture level, non-maize impurity content and mouldy grains in samples from different localities in Benin and Niger.

Country	Localities	Status	n	Grain moisture (%)	Impurities (%)	Moldy grain (%)
Benin	Malanville	Wholesalers	9	11.8 ± 0.1	3.6 ± 0.7	0.3 ± 0.0
		Retailers	9	13.1 ± 0.2	0.9 ± 0.0	0.1 ± 0.0
		T-TEST		*T* = −6.17; df = 1/16; *P* = 0.55	*T* = 3.97; df = 1/16; *P* < 0.01	*T* = 4.99; df = 1/16; *P* < 0.05
	N'Dali	Wholesalers	18	12.5 ± 0.0c	2.5 ± 0.1a	0.9 ± 0.1a
		Retailers	18	13.0 ± 0.0b	0.5 ± 0.1c	0.0 ± 0.0c
		Farmers	18	13.6 ± 0.1a	1.2 ± 0.0b	0.3 ± 0.0b
		Anova		*F* = 123.12; df = 2/51; *P* < 0.01	*F* = 105.03; df = 2/51; *P* < 0.01	*F* = 46.13; df = 2/51; *P* < 0.01
	Bohicon	Wholesalers	6	14.1 ± 0.4c	0.7 ± 0.2a	0.9 ± 0.2a
		Retailers	6	17.5 ± 0.8b	0.1 ± 0.0b	0.3 ± 0.0b
		Farmers	6	22.7 ± 1.2a	0.2 ± 0.0b	0.5 ± 0.0b
		Anova		*F* = 24.46; df = 2/15; *P* = 0.001	*F* = 9.36; df = 2/15; *P* < 0.01	*F* = 9.92; df = 2/15; *P* < 0.01
Niger	Dosso	Wholesalers	6	12.9 ± 0.2	1.8 ± 0.4	0.7 ± 0.1
		Retailers	6	13.3 ± 0.3	0.5 ± 0.0	0.3 ± 0.1
		T-TEST		*T* = −0.83; df = 11; *P* = 0.66	*T* = 3.14; df = 11; *P* < 0.01	*T* = 3.71; df = 11; *P* = 0.88
	Maradi	Wholesalers	5	12.7 ± 0.4	1.8 ± 0.2	0.8 ± 0.2
		Retailers	5	12.8 ± 0.3	0.7 ± 0.1	0.3 ± 0.0
		T-TEST		*T* = −0.11; df = 9; *P*=0.70	*T*=4.92; df = 9; *P*=0.48	T = 2.44; df = 9; *P* = 0.18
Benin		Wholesalers	33	12.6 ± 0.1b	2.5 ± 0.2a	0.7 ± 0.1a
		Retailers	33	13.8 ± 0.3b	0.6 ± 0.0b	0.1 ± 0.0c
		Farmers	24	15.9 ± 0.8a	0.9 ± 0.1b	0.3 ± 0.0b
		Anova		*F* = 11.48; df = 2/87; *P* < 0.01	*F* = 35.45; df = 2/87; *P* < 0.01	*F* = 37.45; df = 2/87; *P* < 0.01
Niger		Wholesalers	11	12.8 ± 0.2	1.8 ± 0.2	0.8 ± 0.1
		Retailers	11	13.0 ± 0.2	0.5 ± 0.1	0.3 ± 0.0
		T-TEST		*T* = −0.62; df = 21; *P* = 0.66	*T* = 5.03; df = 21; *P* < 0.01	*T* = 4.22; df = 21; *P* = 0.16
Both countries	All Localities	Wholesalers	44	12.7 ± 0.1b	2.3 ± 0.2a	0.7 ± 0.0a
		Retailers	44	13.6 ± 0.2 ab	0.6 ± 0.0b	0.1 ± 0.0c
		Farmers	24	15.9 ± 0.8a	0.9 ± 0.1b	0.3 ± 0.0b
		Anova		*F* = 15.12; df = 2/110; *P* < 0.01	*F* = 43.29; df = 2/110; *P* = 0.001	*F* = 45.54; df = 2/110; *P* < 0.01

**Table 3:** Levels of storage insects in maize grain in samples from different localities in Benin and Niger.

Countries	Localities	Status	n	Live insects and larvae in 500 g samples				Total number of insects in 500 g samples
				*P. truncatus*	*S. zeamais*	*C. ferrugineus*	*T. castaneum*	
Benin	Malanville	Wholesalers	9	2.5 ± 0.7	1.2 ± 0.3	0.1 ± 0.1	0.5 ± 0.3	4.7 ± 0.7
		Retailers	9	0.0 ± 0.0	0.1 ± 0.1	0.0 ± 0.0	0.3 ± 0.1	0.4 ± 0.2
		T-TEST		T = 2.76; df = 17; P < 0.05	*T* = 2.91; df = 17; *P* < 0.01	*T* = 1.00; df = 17; *P* = 0.33	*T* = 2.67; df = 17; *P* < 0.05	*T* = 5.95; df = 17; *P* < 0.01
	N'Dali	Wholesalers	18	–	2.1 ± 0.6a	0.3 ± 0.1a	0.8 ± 0.2a	3.4 ± 0.6a
		Retailers	18	–	0.1 ± 0.1b	0.0 ± 0.0b	0.0 ± 0.0b	0.1 ± 0.0b
		Farmers	18	–	0.0 ± 0.0b	0.0 ± 0.0b	0.0 ± 0.0b	0.0 ± 0.0b
		Anova		–	*F* = 13.10; df = 2/51; *P* < 0.01	*F* = 6.54; df = 2/51; *P* < 0.01	*F* = 20.53; df = 2/51; *P* < 0.01	*F* = 28.31; df = 2/51; *P* < 0.01
	Bohicon	Wholesalers	6	–	–	–	–	–
		Retailers	6	–	–	–	–	–
		Farmers	6	–	–	–	–	–
		Anova		–	–	–	–	–
Niger	Dosso	Wholesalers	6	–	1.8 ± 0.6	–	0.3 ± 0.2	2.2 ± 0.5
		Retailers	6	–	0.0 ± 0.0	–	0.0 ± 0.0	0.0 ± 0.0
		T-TEST		–	*T* = 2.30; df = 11; *P* < 0.05	–	*T* = 1.48; df = 11; *P* = 0.17	*T* = 3.99; df = 11; *P* < 0.05
	Maradi	Wholesalers	5	–	3.4 ± 1.7	–	1.2 ± 0.5	4.6 ± 1.4
		Retailers	5	–	0.0 ± 0.0	–	0.2 ± 0.2	0.2 ± 0.2
		T-TEST		–	*T* = 1.69; df = 9; *P* = 0.12	–	*T* = 2.33; df = 9; *P* < 0.05	*T* = 3.04; df = 9; *P* < 0.05
Benin		Wholesalers	33	0.7 ± 0.3a	1.5 ± 0.3a	0.2 ± 0.0a	0.6 ± 0.1a	3.1 ± 0.5a
		Retailers	33	0.0 ± 0.0b	0.1 ± 0.0b	0.0 ± 0.0b	0.1 ± 0.0b	0.2 ± 0.1b
		Farmers	24	0.0 ± 0.0b	0.0 ± 0.0b	0.0 ± 0.0b	0.0 ± 0.0b	0.0 ± 0.0b
		Anova		*F* = 5.59; df = 2/87; *P* < 0.01	*F* = 14.23; df = 2/87; *P* < 0.01	*F* = 6.12; df = 2/87; *P* < 0.01	*F* = 12.92; df = 2/87; *P* < 0.01	*F* = 34.78; df = 2/87; *P* < 0.01
Niger		Wholesalers	11	–	2.5 ± 0.8	–	0.7 ± 0.3	3.3 ± 0.8
		Retailers	11	–	0.0 ± 0.0	–	0.1 ± 0.1	0.1 ± 0.1
		T-TEST		–	*T* = 2.56; df = 21; *P* < 0.05	–	*T* = 2.61; df = 21; *P* < 0.05	*T* = 4.07; df = 21; *P* < 0.05
Both countries	All localities	Wholesalers	44	0.5 ± 0.2a	1.8 ± 0.3a	0.1 ± 0.0a	0.6 ± 0.1a	3.2 ± 0.4a
		Retailers	44	0.0 ± 0.0b	0.1 ± 0.0b	0.0 ± 0.0b	0.1 ± 0.0b	0.1 ± 0.0b
		Farmers	24	0.0 ± 0.0b	0.0 ± 0.0b	0.0 ± 0.0b	0.0 ± 0.0b	0.0 ± 0.0b
		Anova		*F* = 4.79; df = 2/110; *P* < 0.05	*F* = 19.39; df = 2/110; *P* < 0.01	*F* = 5.22; df = 2/110; *P* < 0.01	*F* = 15.86; df = 2/110; *P* < 0.01	*F* = 44.53; df = 2/110; *P* < 0.01

‘‘--’’ no insects.

**Table 4:** Aflatoxin content in maize samples collected from markets in different localities of Benin and Niger.

Localities	Traders	Aflatoxin levels							Total
		0 ppb	0.1–20 ppb	21-100 ppb	101-500 ppb	501-1000 ppb	1001-2500 ppb	2501-3000 ppb	
Malanville	Wholesalers	0	0	0	9	0	0	0	9
	Retailers	0	5	2	2	0	0	0	9
N'Dali	Wholesalers	0	18	0	0	0	0	0	18
	Retailers	6	12	0	0	0	0	0	18
	Farmers	0	18	0	0	0	0	0	18
Bohicon	Wholesalers	0	5	0	0	1	0	0	6
	Retailers	4	2	0	0	0	0	0	6
	Farmers	0	6	0	0	0	0	0	6
Dosso	Wholesalers	0	3	1	1	0	0	1	6
	Retailers	1	5	0	0	0	0	0	6
Maradi	Wholesalers	0	4	1	0	0	0	0	5
	Retailers	2	3	0	0	0	0	0	5
Total		13	81	4	12	1	0	1	112
